# How Do Patients and Doctors Perceive Medical Services for Rare Diseases Differently in China? Insights from Two National Surveys

**DOI:** 10.3390/ijerph17165961

**Published:** 2020-08-17

**Authors:** Shiwei Gong, Dehe Li, Dong Dong

**Affiliations:** 1 Department of Pharmacy Business and Administration, School of Pharmacy, Tongji Medical College of Huazhong University of Science and Technology, Wuhan 430030, China; shwgong@hust.edu.cn (S.G.); d201981456@hust.edu.cn (D.L.); 2 JC School of Public Health and Primary Care, Faculty of Medicine, The Chinese University of Hong Kong, Hong Kong, China; 3 Shenzhen Research Institute of the Chinese University of Hong Kong, Shenzhen 518057, China

**Keywords:** doctor, patient, perception, medical service, rare disease, China

## Abstract

*Background*: Increasing attention is being paid to improve the quality of life of patients with rare diseases in China. However, we are currently unaware of the problems encountered in the medical services of rare diseases from the viewpoints of doctors and patients. This study addressed the differences in the perceived barriers of diagnosis and treatments for rare diseases between doctors and patients in China. *Methods*: Two independent cross-sectional surveys on the perception of Chinese doctors’ and patients’ experiences with rare diseases were launched online between January and February 2018. A non-probability, convenience sampling method was employed to recruit participants. *Results:* In all, 45 rare diseases were reported by 139 doctors and 1853 patients. Patients with rare diseases faced significantly more difficulties in receiving accurate diagnosis (72.0%) and accessing information related to diagnosis and treatment (77.3%) as compared with doctors (34.5% and 40.3%, *p* < 0.0001, respectively). Specially, patients felt more difficulties than doctors in obtaining sustainable treatment for rare diseases (84.3% vs. 49.6%, *p* < 0.001). A higher percentage of patients (58.7%) than that of doctors (39.1%) had concerns in terms of the affordability of drugs. Further, 66.3% patients claimed that the drugs used to treat their conditions were not covered by their current medical insurances, whereas only 21.6% for doctors (*p* < 0.0001). Moreover, 35.3% of doctors responded that they recommended patients to visit the specialist they knew or were acquainted with, whereas 30.0% of patients said that their doctors chose to treat them based on their past experiences (*p* < 0.001). *Conclusion*: The perceived experience of patients with regard to diagnosis and treatment was significantly different from that of doctors. An integrated medical service platform should be established to facilitate better communication and mutual understanding of rare diseases between patients and doctors.

## 1. Background

Rare diseases are often debilitating, disabling, life-threatening, and life-limiting [1]. There is no universal definition of rare diseases globally; however, the threshold of the definitional prevalence varies from 1 to 6.4 per 10,000 [2]. Although each rare disease only affects a small population, the impacts of the total 7000 to 8000 distinct types of rare diseases are significant. In the United States, the total number of people affected by rare diseases has reached 2% to 8% of its population; in Europe, the proportion is 6% to 8% [3,4]. However, in the world’s most populous country, China, there is no available epidemiological data on rare diseases. According to a recent study, rare disease prevalence in Hong Kong is approximately 1.5% [5]. It is therefore plausible to estimate that approximately 21 million people are affected by rare diseases in China.

Approximately 80% of all rare diseases have a genetic origin, but many of them do not have a clearly identifiable cause [6,7]. It is not uncommon for people affected by rare diseases to have diagnostic delays [8]. In the United Kingdom (UK), a report by Limb et al., (2010) from Rare Disease UK (RDUK) showed that 46% of UK rare disease patients were misdiagnosed, after 25% of them had attended either three or four different clinics and 12% had visited more than five different clinics for a definitive diagnosis. Moreover, 46% of patients waited over one year for a definitive diagnosis, with 20% waiting at least five years. After undergoing such a prolonged process for a confirmed diagnosis, 52% of the patients still felt that they had not been provided with sufficient information on their conditions [9]. Similarly, in Australia, a study by Molster et al. showed that 51.2% of patients with rare diseases waited one or more years for a definitive diagnosis, with 30% waiting five or more years; further, 45.9% were misdiagnosed at least once, and 72.1% received inadequate information during diagnosis [10]. For children with rare diseases, Zurynski et al. revealed that the most common problems encountered by pediatricians were diagnostic delays (65%), lack of available treatments (40%), clinical guidelines (36%), and uncertainty in peer support referral (35%) [11].

Diagnosis is only the first step toward the long journey of living with a rare disease. There are only approximately 5% of rare diseases that have a certain treatment, and even in these, the availability, accessibility, and affordability of treatments is an issue. According to a comprehensive review of policies regulating orphan drug accessibility in 35 countries, the availability and affordability of these drugs are often impacted by their high price [12]. As of December 2018, although 74 of the 121 officially recognized rare diseases have drugs available in other countries, China only has drugs available for 53 of these. Among the 83 drugs that can be used to treat these 53 rare diseases, 55 are not off-label (registered with indications for rare diseases, involving 31 rare diseases) and only 29 are included in the Basic National Medical Insurance. In comparison, in the US, EU, and Japan, 162 drugs for all 74 rare diseases are available for patients. Hence, the availability and accessibility of rare disease treatments are of a serious concern in China [13].

Even when these drugs are available and accessible, patient affordability may be a concern. Unlike countries that have a national plan for financing high-cost diseases [12], China does not yet have one. Most treatments must be paid out-of-pocket by the patients. For example, treating Gaucher’s disease requires lifelong enzyme replacement therapy for imiglucerase. In China, the average annual cost of imiglucerase is approximately USD 133,000 (USD1 ≈ CNY7) [14]. Dong and Wang’s (2016) study reported that the medical expenditure of an individual with a rare disease was three times higher than his/her individual income and 1.9 times higher than his/her family income [15]. Xin et al. also estimated that nearly 4.6 million individuals in China suffered from poverty because of their rare conditions [16].

The four barriers faced by rare disease patients—diagnosis, treatment availability and accessibility, and affordability—have previously been well studied in China and other countries. However, the perceptions of medical professionals, especially the doctors who have attended to patients with rare diseases, with regard to such barriers are less explored. Further, even when the doctors’ perceptions are surveyed, they have rarely been compared with those of patients. Nevertheless, these barriers will have a greater impact on patients if their doctors do not perceive them in a similar manner. In real-life situations, the discrepancy between the patients’ and the doctors’ perceptions regarding diseases and treatments may pose a significant influence on the patient–provider collaboration, which in turn will affect patient outcomes. Therefore, our objective in this study is to investigate and compare the perceptions of disease experiences among rare disease patients and doctors who have attended to patients with the same rare diseases. Moreover, we focus on the perceptions of both in terms of rare disease diagnosis, availability and affordability of treatment, and sustainability of medical services.

## 2. Methods

### 2.1. Survey Design

Two cross-sectional national surveys regarding the perception of Chinese doctors and patients on their experiences with rare diseases were launched online (www.wenjuan.com) between January and February 2018. Epidemiological information regarding people affected with rare diseases in China is largely unknown and thus no complete sample frame is present. Therefore, we employed a non-probability, convenience sampling method to recruit participants. In collaboration with the Illness Challenge Foundation (ICF), one of the largest umbrella rare disease patient organizations in China with extensive connections to patients and doctors, the two survey links were circulated among the online and offline networks of the foundation. Recruitment information was also shared by other patient organizations and individuals through snowball sampling. The two surveys were approved by the Medical Ethics Committee of Tongji Medical College of Huazhong University of Science & Technology (Registration No.: S005) and the Committee on the Use of Human and Animal Subjects in Teaching and Research of Hong Kong Baptist University (Registration No.: FRG2/15-16/052).

### 2.2. Questionnaire

Our core research question is: What are the similarities and differences between patients’ and doctors’ perceptions on these issues?

To answer the question, we created the following analytical model to frame the comparison between patients and doctors in four different dimensions: perceived severity of diseases, perceived barriers to diagnosis, perceived barriers to treatment, and perceived referral options. These four dimensions are created for the following reasons:

Perceived severity: Different perceptions on severity of the disease would influence an individual’s motivation to get the disease diagnosed and undergo treatment [17]. Thus, identification of how doctors and patients perceive disease severity is a key step in beginning to understand doctors’ and patients’ perceptions on rare diseases.

Perceived barriers to diagnosis: This dimension was classified into perceived accessibility to diagnosis and accessibility to information during diagnosis. Lack of diagnostic tests to recognize the signs and symptoms of rare diseases would pose an access barrier to diagnosis. Apart from the accessibility to diagnosis, barriers encountered by doctors and patients during the process of gathering disease-related information could lead to perceived barriers to diagnosis.

Perceived barriers to treatment: This dimension was further measured via four sub-dimensions: availability, accessibility, affordability, and continuation of treatment for rare diseases. There is still no effective treatment for most rare diseases. Further, even when drugs are available for some rare diseases, access to orphan drugs becomes an obstacle for patients. Because of the prohibitive costs of drugs, rare disease patients encounter barriers in terms of affordability of drugs. As reimbursement by public funds plays an important role in improving affordability of treatment, the perceptions of reimbursement rates by doctors and patients would influence how doctors and patients perceive affordability of treatment. Because rare diseases are often chronic, requiring prolonged treatment, we included continuation of treatment as one of the components of perceived barriers to treatment.

Perceived referral options: Given the situation of no definitive diagnosis and available treatment, perceptions on referral options (e.g., advice to go home or visit other specialist hospitals) were also investigated and compared between the doctor and patient groups.

Both surveys were primarily conducted online to maximize accessibility by the widespread population. Although the surveys were self-administered, previous studies have indicated that online surveys have more accurate results than telephone surveys [18]. Informed consent was obtained before participants completed the questionnaires. At the beginning of the patient survey, a series of exclusion criteria questions were used to identify the target respondents (i.e., “Are you a patient with rare disease(s)?”; “Are you a caregiver of patient with rare disease(s)?” “What is the name of the rare disease that you (or the patient) have?”). Patients under 18 years of age were directed to the end of the survey and were asked to relay the survey link to their legal guardians. Main caregivers and patients were identified and diverted to two different versions of questionnaires (which covered the same measures, but with questions posed in different ways to retrieve more accurate answers).

In addition to questions tailored for each survey group, eight questions that corresponded to the four comparative dimensions displayed in the analytical model (i.e., perceptions of rare disease diagnosis, information accessibility, treatment accessibility, and referral options) were used in both patient and doctor surveys. These questions were almost identical in both surveys except that in some questions the “subjects” being addressed were different (e.g., in the patient survey, they were asked “what did your doctor do when s/he could not figure out what was wrong with you?”, whereas in the doctor survey, they were asked “what did you do when you could not figure out what was wrong with the patient?”). The purpose of using matched questions in both surveys was to compare the perceptions of patients and doctors with regard to their experiences on key issues related to rare disease diagnosis and treatment, and therefore to find out the cognitive and experiential discrepancies between the two groups.

### 2.3. Sample

In all, 285 doctors and 2040 patients provided valid responses to the two surveys. Among the 285 doctors, 89 reported that they had never encountered any rare disease patients in their career-life. The remaining 196 doctors had collectively encountered 65 rare diseases, whereas the patients were collectively affected by 109 different rare diseases. Between doctors and patients, 45 rare diseases were found to be mutually experienced, which may help narrow down the vast variations in diagnosis and treatment resulted from the different natures of the diseases. Therefore, finally, 139 doctors and 1853 patients, representing the population affected by the 45 mutually experienced rare diseases, were included as the study sample (see Appendix Table A1 for details of the 45 rare diseases). Rare diseases as a whole are too complex and hard to compare. Hence, a valid comparison between the doctors and patients in terms of their perceptions on disease experiences is possible only by excluding patients with rare diseases other than the 45 mutual ones.

### 2.4. Statistical Analysis

Two independent sample T tests and Chi-square tests were used to test differences between the continuous and categorical variables in the survey between the doctor and patient groups using SPSS 13.0 software (SPSS Inc., Chicago, IL, USA). A two-sided test with a *p*-value less than 0.05 was considered to be statistically significant.

## 3. Results

### 3.1. Demographic Characteristics

Table 1 shows the demographic characteristics of the 1853 patients and 139 doctors under comparison. The doctors worked at hospitals from 24 provinces, autonomous regions, and municipalities. The patients were from 32 provinces, autonomous regions, municipalities, and one special administrative region, Macao. Among the doctors, 58.3% were females, whereas among patients, 46.6% were females. Overall, patients were much younger than doctors. Just over 72% doctors were aged 35–64 years, whereas approximately 50% patients were under 18 years of age. Almost 90% doctors were registered at non-rural residences, whereas among the patients, the rural to non-rural household registration ratio was close to 1:1. The 139 doctors had obtained higher educational attainment compared with patients. While all doctors had a college degree or above, only 23.3% patients reported such a level of education.

### 3.2. Perceived Severity of Rare Diseases

While patients tended to rate their diseases to be less severe than doctors, perceptions on the severity of the 45 rare diseases between groups were not statistically different. The mean of patients’ ratings on the severity of their diseases was 7.3 (SD = 2.4), whereas that of doctors was 7.7 (SD = 1.9) (*t* = −1.7, df = 1976, *p* = 0.088).

### 3.3. Perceived Barriers to Diagnosis

#### 3.3.1. Accessibility to Diagnosis

Among patients, 65.5% had been misdiagnosed before receiving a definitive diagnosis. For a definitive diagnosis, 27.2% patients had to visit three or four different hospitals, and over 16% even had to attend five or more different hospitals. With regard to the diagnostic time, 58.8% patients received a confirmed diagnosis during their first year of hospital visits, whereas 20.9% received a confirmed diagnosis after more than two years (for details see Appendix Table A2).

Table 2 shows a comparison between the perception of doctors and patients regarding accessibility to diagnosis on rare diseases. In all, 34.5% doctors and 72.0% patients rated access to diagnosis as difficult; whereas 43.9% doctors and 19.4% patients rated diagnostic accessibility as moderately difficult. (ꭓ^2^ = 85.7, df = 2, *n* = 1978, *p* < 0.001).

#### 3.3.2. Accessibility to Information

Further, Table 2 also presents 40.3% doctors and 77.3% patients have difficulty in accessing diagnostic information. Moreover, 43.2% of doctors and 18.1% patients rated it as moderately difficult. The perceptions of doctors and patients for the accessibility of diagnostic information were significantly different. (ꭓ^2^ = 97.8, df = 2, *n* = 1978, *p* < 0.001).

### 3.4. Perceived Barriers to Treatment

#### 3.4.1. Availability of and Accessibility to Treatment and Drugs

Table 3 shows the perceptions on accessibility to treatment and drugs for rare diseases between the two groups. Only 7.5% doctors and 17.3% patients thought that treatments were not currently available. In other words, more doctors than patients believed that the rare diseases they treated had a certain treatment method (ꭓ^2^ = 20.7, df = 1, *n* = 1992, *p* < 0.001).

Patients reported their mean number of hospital visits to be over 11 per year (SD = 32). They also expressed greater concerns over the sustainability of rare disease treatment. Approximately 84.3% patients felt that continuous treatment access was difficult and 12.7% felt that it was moderately difficult. In comparison, the proportion of doctors who felt that it difficult for patients to be treated continuously was only 49.6%. More than half of the doctors believed that the difficulty of undergoing continuous treatment for patients was average and easy. This difference was statistically significant (ꭓ^2^ = 147.4, df = 2, *n* = 1926, *p* < 0.001).

With regard to treatment of rare diseases, a higher proportion of doctors (17.3%) believed that the 45 rare diseases were currently untreated, compared with only 6.7% of patients with this knowledge (ꭓ^2^ = 20.7, df = 1, *n* = 149, *p* < 0.001). In addition, the treatment methods employed or recommended by doctors and those actually followed by patients were different. According to the doctors, 25.9% of rare diseases were treated with surgery, 69.8% with drugs, and 40.3% with rehabilitation; these proportions reported by patients were 20%, 44.1%, and 13.8%, respectively (details can be seen in Appendix Table A3). Although more doctors reported on the use of drugs to treat rare diseases, fewer doctors (84.3%) than patients (93.9%) thought that these drugs were available (ꭓ^2^ = 13.5, df = 1, *n* = 931, *p* < 0.001).

When dealing with rare diseases, drug treatment remains the primary method. In our surveys, the top three drugs mentioned by doctors for rare diseases were prednisone, pyridostigmine bromide, and vitamin B_12_, whereas those mentioned by patients were hydrocortisone, rapamycin, and methylprednisolone. Further, the proportion of doctors and patients who found drug accessibility to be difficult was 33.7% and 31.2%, respectively, whereas 43.2% and 41.1% of doctors and patients, respectively, found the drug accessibility to be moderately difficult. A higher percentage of patients believed that accessing drugs that they needed was easy (27.8%) compared with the percentage of doctors (23.2%). Overall, the differences in the perception of the difficulty of accessing drugs between doctors and patients were not statistically significant, although there was a tendency showing that the doctors perceived accessibility as relatively more difficult (ꭓ^2^ = 0.9, df = 2, *n* = 801, *p* = 0.635).

#### 3.4.2. Affordability of Drugs

The mean annual medical expense reported by rare disease patients was USD 11,950 (range: USD 7 to USD 714,439, *n* = 1773, SD = 39,218.24). The mean out-of-pocket medical expense paid by patients was USD 9743 (range: USD 0 to USD 714,439, *n* = 1740, SD = 35,296.1). The mean reimbursement ratio of medical insurance for rare disease treatment was 16.8% (range: 0 to 100%, *n* = 1740, SD = 22.2)

Table 4 shows the perceived affordability of drugs for rare diseases between the doctor and patient groups. Among patients, in total, 58.7% rated the affordability of drugs that they used as “bad”, and 35.8% rated it as “acceptable”. In comparison, 39.1% doctors rated the affordability as “bad”, and 48.9% rated it as “acceptable”. More than 10% of doctors thought the affordability was “good”. The perceived affordability of drugs was significantly worse in patients than in doctors (ꭓ^2^ = 14.7, df = 2, *n* = 816, *p* = 0.001).

The issue of affordability was closely associated with the degree of healthcare insurance coverage by the government. When asked whether the drugs they used for the treatment of rare diseases were covered by any government-sponsored medical insurance, 66.3% of patients answered “no”. In comparison, only 21.6% of doctors gave this response. Furthermore, almost a quarter (23.7%) of the doctors who had no clear knowledge about the medical insurance coverage plan, and 31.0% doctors reported that the rare disease drugs were reimbursed by the government at a level higher than 50%, whereas only 9.7% patients reported so. In short, patients reported a significantly lower healthcare insurance coverage rate than doctors (ꭓ^2^ = 85.8, df = 5, *n* = 863, *p* < 0.001).

The doctors’ and patients’ views on the proportion of social or charity donations were also significantly different (ꭓ^2^ = 134.8, df = 5, *n* = 854, *p* < 0.001). While 85.1% of patients reported that the drugs they used for the treatment of their rare diseases were not donated by any pubic or charity groups, 60.8% doctors reported the this. Among the 757 patients who answered this question, only two reported that more than 75% of the expenses of their medications were covered by donations, whereas among the 97 doctors who responded, 16 reported that the drug expense coverage rate was higher than 0% but lower than 75%. More doctors (22.7%) than patients (14.7%) reported that they did not know if there were donations.

### 3.5. Perceived Referral Options

When doctors were unable to diagnose the disease the patients had or when there were no treatment options for patients, they would usually provide referral advice to the patients. The differences in referral recommendations reported by the doctors and the patients are shown in Table 5.

When making referrals, 35.3% of doctors chose to recommend the patient to visit the specialist they knew or were acquainted with, whereas 31.7% would advise the patient to visit a specialist they had only heard of. Some doctors would not mention “names”, but rather recommend the patient to transfer to other higher-level hospitals in other cities (25.9%), visit other specialist hospitals they knew quite well (23.7%), or visit the specialist hospitals that the doctor had just heard of (18.7%). Only 7.2% of doctors claimed that they would treat the patients based on their past experiences. No doctor reported that they would advise their patients to go home.

However, according to patient survey results, 26.6% patients reported that they were advised by their doctors to go home when a diagnosis could not be made or a treatment could not be provided. Moreover, 30.0% of patients said that their doctors chose to treat them based on their past experiences. With regard to making referrals, patients reported that 23.3% of their doctors recommended them to visit a higher-level hospital in other cities, 9.8% doctors recommended them to visit the specialist doctor of whom they had heard of, and 8.0% doctors recommended them to visit the specialist hospitals that they had heard of.

## 4. Discussion

Our study was the first to explore the differences in perception between doctors and rare disease patients with regard to medical services for 45 rare diseases through two national surveys in China. These findings unveil the magnitude of these differences allowing us to consider the implications of the discrepancies in perceptions between the two groups.

While the stark differences in perception between these two groups may be attributable to factors other than patients and doctors not seeing eye to eye (e.g., these patients and doctors do not have direct connections), it is undeniable that the statistical significance found for differences in most factors in the survey warrants further examination. One of the most important findings here was revealing how doctors really do not see how hard this is on their rare disease patients. Patients perceived greater difficulty than doctors in having definitive diagnosis, accessing disease-related information, being provided with available treatments, or affording the prescribed drugs. The burden of these factors lies more on the patients themselves than on the doctors who are trying to diagnose and treat them. While a doctor may only encounter one or two patients with rare diseases in their lifetime, their patients not only live with the disease but also continuously deal with its challenges. Awareness needs to be raised among doctors who treat these patients so that they better understand how their patients perceive all aspects of their condition. One important avenue to deal with the difficulties faced by these patients are rare disease patient organizations.

A patient organization is a non-profit organization and comprises patients with a particular disease with a mission to advocate for patients’ interest at the personal level, as well as the policy level, and to assist patients by providing services and resources that may not otherwise be available. Patient organizations allow individuals who are marginalized by their illness to have a say in the decision-making process when it comes to addressing their particular disease. For those with rare diseases, these organizations can give a stronger voice to patients dealing with something that most others are unaware of. Rare disease patient organizations have been developing in China since around the early 2000s. While they have substantially grown since then, these organizations still face several challenges in China such as, relatively few numbers of organizations, low level of organizational specialization, lack of organizational stability, limited social influence, and limited ability to access social resources [13]. Building strong patient organizations is vital to improving the circumstances of rare disease patients. These issues must be addressed to provide more adequate support to patients with rare diseases. Patient organizations can be a key player in advocating improvements in all of the issues presented in this study.

Patients and doctors alike acknowledged difficulties in reaching a definitive diagnosis and implementing a sustainable treatment plan. However, in reality, when such difficulties unsurfaced, the doctors might try to treat the patients based on their own experience or refer the patients to someone or somewhere else. Referral constitutes a critical link to rare disease patients’ diagnosis and treatment [19] and has deep influence on the patients’ health. However, not all doctors are aware that their decisions on referral might have such an influence. As our surveys reveal, there was a clear discrepancy in how doctors and patients perceived referral options. All these discrepancies point to the lack of a clear and standardized protocol to provide detailed guidance on medical services for rare diseases, including diagnosis, referral, counselling, and treatment.

In May 2018, China released the First National List of Rare Diseases comprising 121 rare diseases [20]. This list is meant to help better equip medical professionals to treat these diseases as well as develop drugs effective for treating them. Time is needed to assess the effectiveness of this new effort for addressing rare diseases. After discovering that general practitioners had a low level of awareness and knowledge of rare diseases, a study in Belgium recommended revision to rare disease education in medical schools, focusing on the “red flags” of rare diseases among general practitioners, as well as providing a more universal database to inform on symptoms, diagnostic tests, and available reference centers to all doctors [21]. Although it looks like China has taken a step in this direction for creating the national database, more practical measures should be taken to address the immediate needs of rare disease patients such as teaching doctors to look out for “red flags” of rare diseases to shorten the time to diagnosis; further, a standardized referral method could also help reduce confusion of patients in terms of referrals. Zhou and Nunes found three core issues in terms of knowledge-sharing among Chinese healthcare referrals: (1) the absence of national and local policies for inter-hospital knowledge-sharing; (2) lack of a specific hospital knowledge-sharing requirement; and (3) lack of mutual acquaintance. These must also be addressed to develop more timely diagnoses of rare diseases [22].

Our study also found that 65.5% of patients with rare diseases had been misdiagnosed before receiving a definitive diagnosis in the survey, which is much higher than the proportions in the United Kingdom (46.0%) [9] and Australia (45.9%) [10]. In primary hospitals or rural hospitals in China, most doctors did not have proper diagnostic equipment for rare diseases or were aware of standard treatment guidelines. Therefore, the greater lack of professional knowledge about rare diseases or the absence of knowledge-sharing policy and platform might be one of the main reasons underneath the high proportion of misdiagnosis in China. To change such a situation, regular special continuing education training programs and peer experience sharing system are needed to be established in the future in China.

Both doctors and patients in this study believed that lack of affordability was a big obstacle in treating rare diseases. Previous studies by Dong and Xin have shown that patients with rare diseases in China encounter very high medical costs and unaffordable financial burdens [15,16]. A survey by Bin et al. also found the average annual medical expenditure of patients affected by seven rare diseases in Fujian Province to be USD 15,575, with the maximum being USD 314,353, in 2015. The biggest difficulty faced by the majority of these patients during treatment was high treatment cost. Further, the out-of-pocket cost exceeded 40.0% of annual family income among 77% of patients [23]. Gong et al. found that within a periodic treatment course, the average treatment cost using 23 orphan drugs equated to 505.6 days of per capita net income for a middle class urban resident or 1582.8 days of income for a rural middle class resident in China [24]. Fei’s study found that the drug list of China’s National Basic Medical Insurance (2017 edition) covered 53 orphan drugs, of which 19 orphan drugs were covered in the Class A list (zero out-of-pocket ratio), covering 11 rare diseases. Further, the Class B list (30% out-of-pocket ratio) contained 34 orphan drugs, covering 13 rare diseases [25]. Thus, rare disease drug coverage continues to be poor and limited. Poor affordability of drugs and treatment decrease the quality of life not only for the patient but also for the family, and may pull the entire family into poverty trying to cover medical costs. Doctors need to be even more aware of this burden when considering treatment options for their patients; they should also themselves advocate to increase the affordability of the necessary drugs to treat their patients. In addition, China has a very unevenly distributed system of high-quality medical services. It is very difficult to find high-quality medical services for patients with rare diseases who are widely scattered across the country. Patients have to spend for a lot of money and time to seek information and find a definitive diagnosis for the disease they have. Even if they find it, accessibility and affordability to treatments, even if just the symptomatic ones, are still challenges. The lack of incentive policies for new orphan drugs, over-reliance on imported orphan drugs, and no medical insurance compensation plan for orphan drugs and rare diseases have forced the patients to stop active treatment. It is therefore imperative for China to issue professional guidelines and standardized protocols for doctors and patients so that they could have continuous access to medical services for rare diseases.

## 5. Study Limitations

The study has several limitations. First, due to the limitation of resources, the patients and the doctors were sampled through snowballing, which endangers the representation and generalizability of our findings. Second, the doctors and the patients were not true pairs in real life; rather, they were matched based on disease. Since many doctors might have experience with multiple diseases, whereas patients mainly had experience with one disease, their perceptions of the difficulties involved in rare disease diagnosis and treatment might differ. Third, since there are very few rare diseases that have curative treatment, affordability to available treatments was measured mainly based on symptomatic therapies or the lack of evidence-based treatments. Therefore, the perception of the affordability of rare diseases was not reflecting the patients’ or the doctors’ understanding of curative treatments for rare diseases, which can be much more expensive than the symptomatic ones.

## 6. Conclusions

Patients with rare diseases in China clearly have a different perception from doctors in terms of difficulty in accessing accurate diagnosis, disease-related information, sustainable treatment, affordability, and clear referral paths. Thus, a national plan for rare diseases in China should be supported. This should include establishing a specialized information platform of treatment centers for diagnosis and treatments of rare diseases with clinical standard guidelines, improving the medical insurance system specified for rare diseases, and strengthening education and training of healthcare professionals at different levels to provide definitive diagnosis, treatments, and referral paths to patients with rare diseases. Future research should pay more attention on how to strengthen the communication between doctors and patients and explore successful medical services for improving the quality of life of these patients.

## Figures and Tables

**Table 1:** Demographic characteristics of surveyed doctors and patients.

Characteristics	Doctor Group *n* (%)	Patient Group *n* (%)	*n*
Total number	139	1853	1992
Gender			1992
Female	81 (58.3)	863 (46.6)	
Male	58 (41.7)	990 (53.4)	
Age groups			1990
<18 years	0	865 (46.7)	
18–34 years	36 (26.1)	515 (27.8)	
35–64 years	100 (72.5)	457 (24.7)	
≥65 years	2 (1.5)	15 (0.8)	
Household Registration			1992
Non-rural Residence	125 (89.9)	964 (52.0)	
Rural Residence	12 (8.6)	881 (47.0)	
Others	2 (1.4)	8 (0.4)	
Education level			1990
Up to secondary school	0	1421 (76.7)	
College *	4 (2.9)	186 (10.0)	
Undergraduate	29 (21.0)	203 (11.0)	
Postgraduate and higher	105 (76.1)	42 (2.3)	

* Three-year college or vocational school.

**Table 2:** Perceptions on accessibility to diagnosis of and information on rare diseases between groups.

Items	Doctor Group *n* (%)	Patient Group *n* (%)	ꭓ^2^ (Between Group *p*-Value)	*n*
Accessibility to diagnosis			85.7 (<0.001)	1978
Difficult	48 (34.5)	1324 (72.0)		
Moderately difficult	61 (43.9)	356 (19.4)		
Easy	30 (21.6)	159 (8.6)		
Accessibility to information			97.8 (<0.001)	1978
Difficult	56 (40.3)	1421 (77.3)		
Moderately difficult	60 (43.2)	332 (18.1)		
Easy	23 (16.5)	86 (4.7)		

**Table 3:** Perceptions on the availability and accessibility to treatment and drugs for rare diseases between the two groups.

Items	Doctor Group *n* (%)	Patient Group *n* (%)	ꭓ^2^ (Between Group *p*-Value)	*n*
Availability of treatment ^a^			20.7 (<0.001)	1992
No	24 (7.5)	125 (17.3)		
Yes	115 (92.5)	1728 (82.7)		
Difficulty in accessing sustainable treatment			147.4 (<0.001)	1926
Difficult	66 (49.6)	1512 (84.3)		
Moderate	37 (27.8)	227 (12.7)		
Easy	30 (22.6)	54 (3.0)		
Availability of drugs			13.5 (<0.001)	931
No	18 (15.7)	50 (6.1)		
Yes	97 (84.3)	766 (93.9)		
Difficulty in accessing drugs			0.9 (=0.635)	801
Difficult	32 (33.7)	220 (31.2)		
Moderate	41 (43.2)	290 (41.1)		
Easy	22 (23.2)	196 (27.8)		

^a^ available treatment includes drugs, surgery, rehabilitation and other therapies for rare diseases.

**Table 4:** Perceived payment levels of drugs for rare diseases between the two groups.

Items	Doctor Group *n* (%)	Patient Group *n* (%)	ꭓ^2^ (Between Group *p*-Value)	*n*
Affordability to drugs			14.7 (=0.001)	816
Bad	36 (39.1)	425 (58.7)		
Acceptable	45 (48.9)	259 (35.8)		
Good	11 (12.0)	40 (5.5)		
Drug coverage rate (CR) ^a^			85.8 (<0.001)	863
CR = 0	21 (21.6)	508 (66.3)		
0% < CR < 25%	9 (9.3)	41 (5.4)		
25% ≤ CR < 50%	14 (14.4)	34(4.4)		
50% ≤ CR < 75%	9 (9.3)	31 (4.1)		
CR > 75%	21 (21.7)	43 (5.6)		
Not clear	23 (23.7)	109 (14.2)		
Drug donation rate (DR) ^b^			134.8 (<0.001)	854
DR = 0	59 (60.8)	644 (85.1)		
0 < DR < 25%	11 (11.3)	0		
25% ≤ DR < 50%	2 (2.1)	0		
50% ≤ DR < 75%	3 (3.1)	0		
DR > 75%	0	2 (0.3)		
Not clear	22 (22.7)	111 (14.7)		

^a^ Coverage rate (CR) of drugs by government-sponsored medical insurance; ^b^ donation rates (DR) of drugs from society.

**Table 5:** Referral options when there is no definitive treatment for rare diseases between the two groups.

Items	Answer	Doctor Group	Patient Group	ꭓ^2^	Total No.
	Yes/No	*n* (%)	*n* (%)	(Between Group *p*-Value)	
Treat patients according to doctor’s past experience	Yes	10 (7.2)	556 (30.0)	33.1 (<0.001)	566
Advise to go home	Yes	0 (0.0)	493 (26.6)	(Fisher’s exact test <0.001)	493
Recommend higher-level hospitals in other cities (but do not mention hospital or doctor names)	Yes	36 (25.9)	432 (23.3)	0.5 (=0.488)	468
Recommend specialist whom the doctor has heard of	Yes	44 (31.7)	181 (9.8)	61.8 (<0.001)	225
Recommend specialist hospitals that the doctor has heard of	Yes	26 (18.7)	148 (8.0)	18.6 (<0.001)	174
Recommend specialist whom the doctor is acquainted with	Yes	49 (35.3)	106 (5.7)	157.1 (<0.001)	155
Recommend higher-level hospitals in the local region	Yes	25 (18.0)	108 (5.8)	30.7 (<0.001)	133
Recommend specialist hospitals that the doctor is acquainted with	Yes	33 (23.7)	80 (4.3)	91.2 (<0.001)	113
Recommend higher-level doctors in the same hospital	Yes	24 (17.3)	69 (3.7)	53.3 (<0.001)	93

## Data Availability

The data that support the findings of this study are available from the corresponding author upon reasonable request.

## Abbreviations

UK	United Kingdom
RDUK	Rare Disease UK
ICF	Illness Challenge Foundation

## Appendix

**Table A1:** Types of rare diseases reported from doctors and patients in the survey.

Disease Name	Doctor Group		Patient Group		Total	
	*n*	%	*n*	%	*n*	%
Albinism	8	4.40	37	2.00	45	2.20
Behcet’s disease (BD)	1	0.50	166	9.00	167	8.20
Osteogenesis imperfecta (OI)	16	8.70	88	4.70	104	5.10
Bullous Epidermolysis (EB)	1	0.50	5	0.30	6	0.30
Multiple Sclerosis (MS)	1	0.50	56	3.00	57	2.80
Fabre disease	4	2.20	80	4.30	84	4.10
Pulmonary hypertension (PAH)	2	1.10	54	2.90	56	2.80
Peritoneal pseudomyxoma (PMP)	2	1.10	65	3.50	67	3.30
Hepatolenticular Degeneration (HLD)	5	2.70	10	0.50	15	0.70
Gaucher disease (GD)	3	1.60	59	3.20	62	3.00
Black spot polyp syndrome (PJS)	1	0.50	81	4.40	82	4.00
Amyotrophic lateral sclerosis (ALS)	17	9.30	3	0.20	20	1.00
Spinocerebellar ataxia (SCA)	1	0.50	10	0.50	11	0.50
Spinal muscular atrophy (SMA)	10	5.50	209	11.30	219	10.80
Spina bifida	1	0.50	2	0.10	3	0.10
Methylmalonic acidemia	18	9.80	1	0.10	19	0.90
Tuberous sclerosis (TSC)	2	1.10	175	9.40	177	8.70
Progressive muscular dystrophy	13	7.10	47	2.50	60	2.90
Lymphangiomyomatosis (LAM)	3	1.60	3	0.20	6	0.30
Meow Syndrome	2	1.10	1	0.10	3	0.10
Nieman Pick Disease (NPD)	1	0.50	16	0.90	17	0.80
Mucopolysaccharidosis (MPS)	3	1.60	109	5.90	112	5.50
Urea cycle disorder	3	1.60	3	0.20	6	0.30
Noonan syndrome	1	0.50	1	0.10	2	0.10
Pompeii (GSD II)	3	1.60	93	5.00	96	4.70
Prader-Willi syndrome	5	2.70	55	3.00	60	2.90
Cartilage dysplasia I	2	1.10	0	0.00	2	0.10
Cartilage dysplasia II	0	0.00	5	0.30	5	0.20
Neurofibromatosis (NF)	2	1.10	38	2.10	40	2.00
Adrenal insufficiency	1	0.50	19	1.00	20	1.00
Adrenal hyperplasia (CAH)	4	2.20	149	8.00	153	7.50
Growth hormone deficiency (GHD)	2	1.10	2	0.10	4	0.20
Optic neuromyelitis (NMO)	1	0.50	48	2.60	49	2.40
Retinoblastoma	1	0.50	1	0.10	2	0.10
Glycogen storage disease	2	1.10	1	0.10	3	0.10
Turner syndrome (TS)	3	1.60	1	0.10	4	0.20
Angel syndrome	1	0.50	3	0.20	4	0.20
Systemic sclerosis (SSc, scleroderma)	2	1.10	41	2.20	43	2.10
Mitochondrial disease	7	3.80	6	0.30	13	0.60
Hemophilia	4	2.20	7	0.40	11	0.50
Hereditary hemorrhagic telangiectasia	1	0.50	1	0.10	2	0.10
Metachromatic leukodystrophy (MLD)	1	0.50	1	0.10	2	0.10
Ichthyosis	3	1.60	1	0.10	4	0.20
Myasthenia gravis (MG)	13	7.10	6	0.30	19	0.90
ANCA-associated vasculitis	1	0.50	2	0.10	3	0.10
GH Tumor/Acromegaly	5	2.70	92	5.00	97	4.80
Total	183	100	1853	100	2036	100

**Table A2:** Patients experience of diagnosis for rare disease.

Items	No. Patients (%)
Misdiagnosis (*n* = 1839)	1214 (65.5)
Diagnostic time ^a^ (*n* = 1814)	
<1 year	1066 (58.8)
1–2 years	370 (20.4)
3–5 years	148 (8.2)
≥6 years	230 (12.7)
No. of hospital visited (*n* = 1821)	
≤2	1032 (56.7)
3–4	496 (27.2)
≥5	293 (16.1)

^a^ Years of disease symptoms onset to confirmed diagnosis.

**Table A3:** Perceptions on treatment types for rare diseases and sources of drugs between two groups.

Items	Doctor Group *n*(%)	Patient Group *n*(%)	ꭓ^2^ (*p*-Value)	*n*
Treatment (multiple choice questions)	139	1853		1992
surgery	36(25.9)	371(20.0)	344.5 (0.000)	407
drug	97(69.8)	818(44.1)	258.5 (0.000)	915
Rehabilitation	56(40.3)	256(13.8)	193.5 (0.000)	312
other	60(43.2)	37(2.0)	90.3 (0.000)	97
No treatment	24(17.3)	125(6.7)	20.7 (0.000)	149
